# Supplementary material for: Tear Cytokine Changes up to One Year After Allogeneic Hematopoietic Stem Cell Transplant: Effect of Daily Topical Cyclosporine-A 0.1% Emulsion
Source: Int J Mol Sci. 2025 Jun 19;26(12):5915. doi: 10.3390/ijms26125915 (PMC12193220; doi:10.3390/ijms26125915)
Supplement: Supplementary file 1 [file ijms-26-05915-s001.zip › ijms-3610336-supplementary.pdf]

Supplementary Files

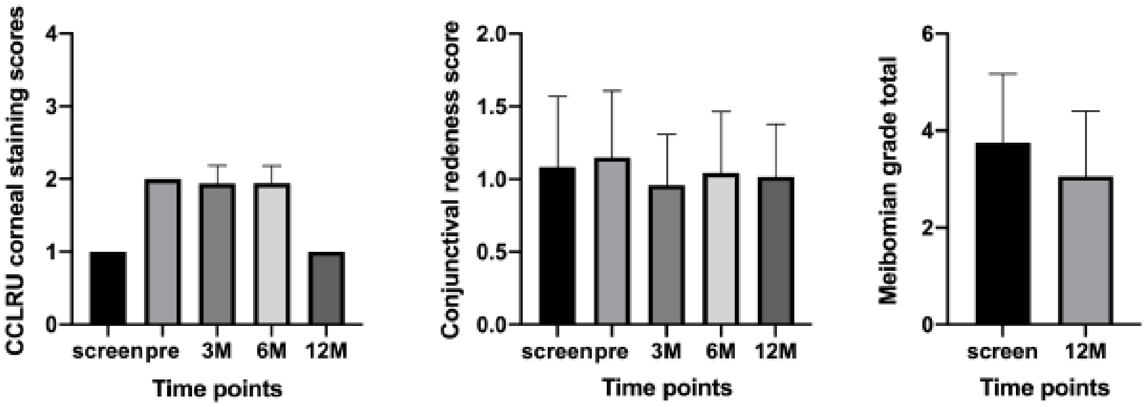

Figure S1. Bar charts showing results of clinical evaluation of all study participants. A. Corneal fluorescein staining scores (total of five corneal zones), B. Conjunctival redness scores, C. Meibomian gland assessment scores. Height of bars represent means and error bars represent standard deviations.

Table S1. patient visit schedule and procedures.

Tests/Consult	Pre – Ikervis (Screening Visit) (Day 0)	Pre – HSCT (4weeks±1 week)	3 months post HSCT (±1week)	6 months post HSCT (±1week)	Ad-hoc visit (not restricted in time)	1 year post HSCT (±2weeks)
Consult	Informed consent, SLE,VA/IOP, fundal exam, MG Evaluator	SLE (if needed), MG Evaluator (optional)	VA/IOP, SLE (if needed), MG Evaluator	VA/IOP, SLE (if needed), MG Evaluator	VA/IOP, SLE MG Evaluator	VA/IOP, SLE, MG Evaluator Fundal exam
SPEED questionnaire	✓	✓	✓	✓	✓	✓
Osmolarity	✓	✓(optional)				
Conjunctival Redness	✓	✓(optional)	✓	✓	✓	✓
NIKBUT-Oculus	✓	✓(optional)	✓	✓	✓	✓
Lipiview	✓					✓
Meibography	✓					✓
Corneal Staining Photo-Oculus	✓	✓(optional)	✓	✓	✓	✓
Schirmer test – cytokine analysis	✓	✓	✓	✓	✓	✓

Impression Cytology		✓			✓ done only at onset of dry eye symptoms	✓ only done if not done at ad hoc visit
Quality of Life questionaire						✓

HSCT: hematopoietic stem cell transplantation; SLE: slit lamp examination; VA: visual acuity; IOP: intraocular pressure; MG meibomian gland; NIKBUT: non-invasive keratograph tear break up time; SPEED: standard patient evaluation of eye drynes.
